# Early-life gut and oral microbiota development: a multi-niche study including mother-partner-infant triads

**DOI:** 10.1186/s12866-025-04521-3

**Published:** 2025-11-15

**Authors:** Kotryna Simonyté Sjödin, Andreas Sjödin, Patrik Rydén, Ingrid Mogren, Magnus Domellöf, Pernilla Lif Holgerson, Christina E. West

**Affiliations:** 1 Department of Clinical Sciences, Pediatrics, Umeå University, Umeå, Sweden; 2 Department of Clinical Microbiology, Umeå University, Umeå, Sweden; 3 Department of Mathematics and Mathematical Statistics, Umeå University, Umeå, Sweden; 4 Department of Clinical Sciences, Obstetrics and Gynecology, Umeå University, Umeå, Sweden; 5 Department of Odontology, Pediatric dentistry, Umeå University, Umeå, Sweden

**Keywords:** NorthPop, Gut microbiota, Oral microbiota, Infant, Vaginal delivery, Caesarean section

## Abstract

**Background:**

Early gastrointestinal microbiota establishment is crucial for host metabolism and immune development, with delivery mode and breastfeeding playing key roles. Vaginal delivery promotes colonization by maternal vaginal and gut microbes, while Caesarean section delivery leads to exposures of environmental and skin-derived microbiota. Although maternal contributions have been studied, the role of paternal exposure in shaping infant microbiota remains underexplored. We hypothesized that both parents influence infant microbiota establishment and therefore investigated the contributions of maternal and paternal microbes, as well as delivery mode, on infant oral and fecal microbiota within 48 h of birth and at 1 month of age.

**Methods:**

We analysed the gut and oral microbiota of 264 pregnant women, 261 partners, and 266 infants using 16S rRNA gene amplicon sequencing. α-diversity (Shannon Index) was compared using Wilcoxon tests, and β-diversity (Bray–Curtis dissimilarity) was assessed with PERMANOVA and PERMDISP. Principal component analysis (PCA) based on centered log-ratio (CLR)-transformed genus-level data was used for ordination and visualisation of taxonomic structure. Differentially abundant taxa across niches and delivery modes were identified using Kruskal–Wallis and Wilcoxon tests with false discovery rate (FDR) correction, followed by linear discriminant analysis (LDA). Putative amplicon sequence variant (ASV) sharing between infants and family members was explored using tree-based phylogenetic plots showing taxon presence and relative abundance across sample types. All analyses were performed in R using established packages.

**Results:**

Adults showed significantly higher microbial α-diversity than infants in both gut and oral samples. β-diversity analyses revealed distinct microbial community structures influenced by ecological niches and delivery mode. Within the first 48 h after birth, differential abundance analyses identified *Lactobacillus crispatus* in meconium and *Blautia_A* in oral swabs enriched in vaginally delivered infants. *L. crispatus* also emerged as a key marker of the vaginal microbiota in our cohort-wide comparison, while *Blautia*, typically a gut-associated genus, was also detected in parental rectal and meconium samples. This co-occurrence may reflect transient microbial seeding during vaginal delivery. However, due to the limited resolution of 16S rRNA gene sequencing, these patterns suggest ecological overlap rather than definitive evidence of vertical transmission.

**Conclusions:**

Our findings demonstrate that delivery mode influences early gut and oral microbiota composition, with vaginal delivery associated with taxa also found in maternal samples. While we observed microbial continuity between infant and parental niches, we could not clearly distinguish partner-specific contributions—likely due to the limited resolution of 16S rRNA gene sequencing. These results highlight the importance of delivery-associated exposures in early microbial development and underscore the need for high-resolution approaches to better resolve microbial acquisition within families.

**Supplementary Information:**

The online version contains supplementary material available at 10.1186/s12866-025-04521-3.

## Introduction

The microbiota of the gastrointestinal (GI) tract, from the oral cavity to the rectum, are critical in host physiology [1, 2] and early colonization parallels the development of host metabolism and immune functions [3–7]. Aberrant colonization patterns of the gut have been associated with the development of early onset non-communicable diseases, thus underscoring the importance and long-term effects of colonization in early life [8]. Notably, emerging research also suggests that oral microbial programming is associated with systemic diseases [9, 10]. Ecological succession with compositional and functional changes of the GI microbiota over the first years of life eventually results in a relatively stable microbial community [11, 12] and this process is driven by interactions between environmental exposures, early diet, microbe-associated and host-related factors [8]. Delivery mode has a considerable influence on the establishment of both the oral [13] and gut [14] microbiota. The first microbial exposures of vaginally delivered (VD) infants are derived from vaginal and perianal microbiota. Consequently, VD infants are initially colonized by microbes from the vagina, gut, and skin [15] whereas Cesarean section (CS) delivered infants typically obtain environmental and skin microbes. Also, the gradual acquisition of a more complex gut microbiota is slower in CS delivered infants than in VD infants [14]. The very early colonization of the infant gut in VD full term infants is dominated by aerobic and facultative bacteria [16]. As oxygen is consumed, conditions become favourable for the growth of anaerobic bacteria, and this process is independent of diet. Once breastfeeding is established, it will result in a gut microbiota rich in bifidobacteria, as human milk is abundant in bioactive components such as human milk oligosaccharides that are specifically suited to promote healthy gut colonization [17].

Although human milk [17] and vaginal and faecal microbiota [15] influence the establishment of the infant GI microbiota, a large part of the infant bacterial community in the GI tract is estimated to be derived from unknown sources. Until recently, the contribution of microbial exposures from the fathers [18] have been largely ignored.

We hypothesized that in addition to the mother, the partner also contributes to the early life GI microbiota establishment in the offspring. We focused on the impact of maternal and paternal (or co-parental) exposures, including human milk, on infant oral and fecal microbiota establishment at two early time points, i.e., within 48 h of birth and at 1 month of age.

## Methods

### Study participants

**Fig. 1:**
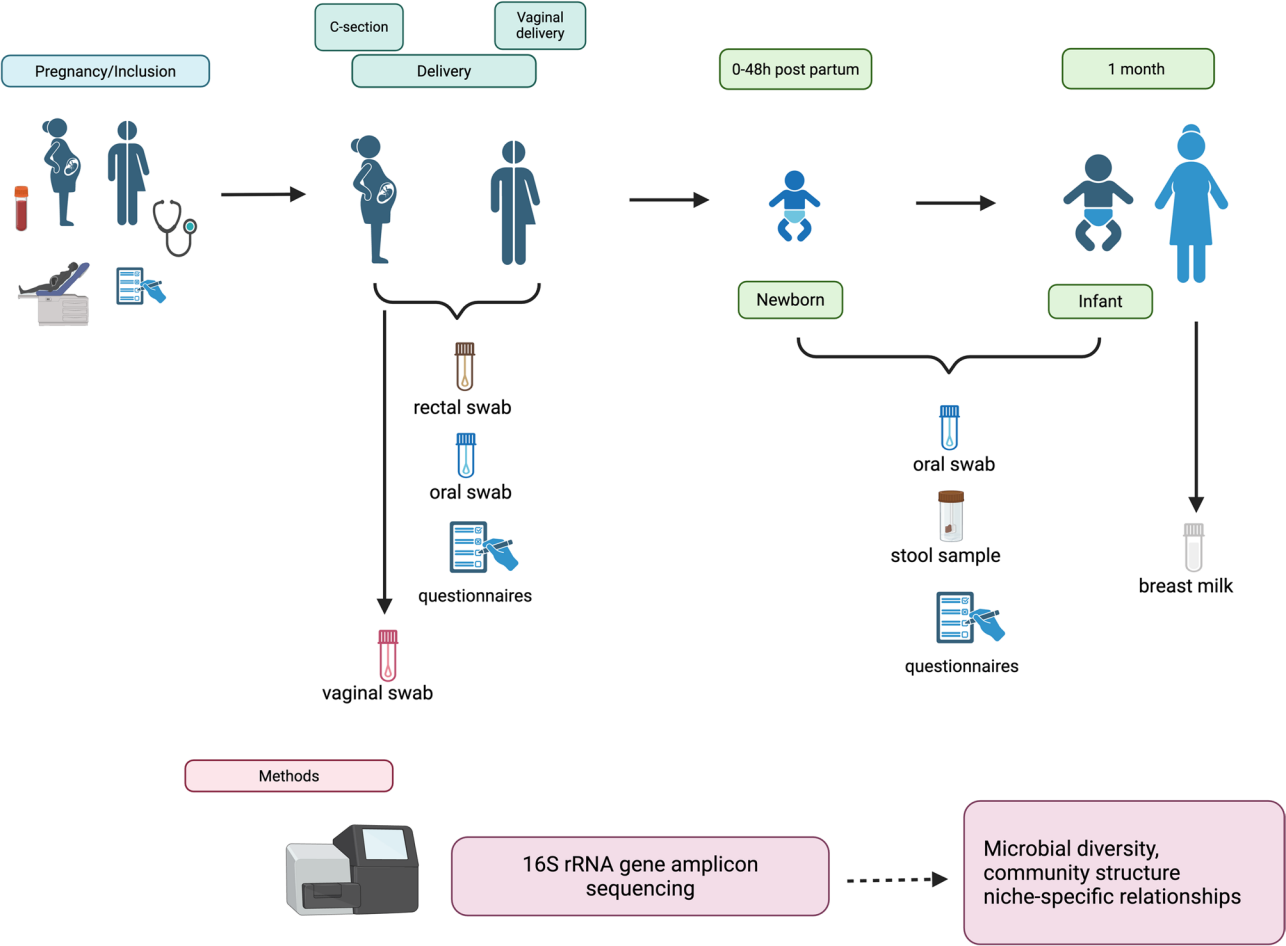
Study overview and sample collection timeline in the FLORA cohort. This figure provides an overview of the FLORA study, a substudy of the NorthPop Birth Cohort in Västerbotten County, Northern Sweden. Parents were invited to participate during the routine ultrasound examination at 14–24 weeks of gestation. Inclusion criteria required pregnant women to be ≥ 18 years of age. A total of 264 pregnant women, 261 partners, and 266 full-term infants were enrolled. Biological samples were collected from mother–partner–infant triads. At the time of hospital admission for delivery, mothers and partners performed self-sampling of vaginal, rectal, and oral swabs (mothers) and rectal and oral swabs (partners). Within 48 h of birth, meconium and oral swabs were collected from infants by the parents under clinical supervision. At one month of age, parents collected a second infant fecal sample, oral swab, and a human milk sample at home. Demographic, environmental, and anthropometric data from parents were collected using web-based self-reported questionnaires during pregnancy. Clinical data for infants, including birth outcomes and neonatal care, were obtained from national quality registers and medical records. Microbial DNA was extracted from all biological samples and sequenced using 16S rRNA gene amplicon sequencing (Illumina MiSeq). Microbial diversity, phylogenetic relationships, and taxonomic biomarkers were analysed across time points and sample types

The FLORA Study is a substudy of the NorthPop Birth Cohort Study (NorthPop), which is an ongoing prospective population-based study in Västerbotten County in Northern Sweden [19]. Parents are invited to participate at the time of the routine ultrasound examination at gestational age 14–24 weeks. Inclusion criteria were: pregnant women ≥ 18 years of age, comprehending the Swedish language, a viable pregnancy at gestational age of 14 to 24 weeks, with an intention to give birth and to reside in the catchment area in the forthcoming years. From October 2018 to February 2021, pregnant women, and their partners in the larger Umeå area that had given informed consent to participate in NorthPop with their child (*n* = 2112), were invited to participate in the FLORA study, which entailed additional oral, fecal and vaginal samplings of the pregnant woman and oral and fecal samplings of the partner. The study procedures are depicted in Fig. 1.

### Data collection

Data were collected using web-based self-reported questionnaires including items on demographic and background characteristics of the parents at gestational age 14–24, 26, 34, and 35 weeks, respectively. Data on gestational age, delivery mode, infant sex, birth weight and birth length, and information about neonatal care, were obtained from the Swedish Neonatal Quality register (SNQ) [20], the Swedish Pregnancy Register [21] and from medical records.

### Collection of biological samples

Pregnant women and their partners self-collected samples on the day of admission to the delivery unit at Umeå University Hospital, prior to delivery: vaginal, rectal and oral swabs from the pregnant woman, and rectal and oral swabs from the partner. Parents collected newborn meconium samples within 0–48 h of birth after receiving instruction and under staff supervision; because meconium passage and clinical routines vary, collection times within this window differed across infants. Newborn oral swab was collected at the time of the Newborn Blood Spot Screening (around 48 h after birth). At 1 month, parents collected an infant fecal sample and oral swab, and provided a human milk sample collected at home.

Floqswabs^®^ (608CS01R, Copan Italia, Italy) were used for vaginal and rectal swabs. The swab was inserted ~ 4–5 cm in the vagina or rectum, respectively, and rotated for approximately 10–30 s. After sampling, the Floqswab was placed in an eNAT^®^ tube containing a medium that preserves and stabilizes the microbial nucleic acids. Sterile cotton bud applicators (20–1192, Applimed SA, Switzerland) were used for oral swabs from both parents and infants. Mothers were asked not to feed the infant during an hour before sampling. The tongue and the inside of the cheek was swabbed, after sampling the applicator was swirled in a tube with 100ul TE buffer (10 mM Tris, 1 mM EDTA, pH 8). The procedure was repeated once with a new applicator which was swirled in the same tube. Meconium or faeces, approximately 2 g, were collected in sterile faeces tubes with a spoon attached to the screwcap (80.734, Sarstedt AG &Co., Germany). Human milk was sampled at home at the first feeding in the morning, when the child was 1 month old. Human milk was expressed either manually or with a breast milk pump and collected into a sterile 50 mL tube. All samples were kept at −20 °C until delivered to the Northern Sweden Biobank where they were stored at −80 °C until analysis.

### Bacterial DNA extraction

DNA was extracted using different commercially available kits depending on the sample type, with the aim of optimising yield and purity for matrices with varying biomass and inhibitory content. Low-biomass oral swabs from infants were processed with a kit optimised for such material [22], while high-biomass matrices such as stool and meconium were processed with bead-beating–based protocols to ensure efficient lysis.

### From vaginal, rectal, and oral swabs, and human milk

We used QIAamp DNA Microbiome Kit (#51704; Qiagen GmbH, Germany) for isolation of bacterial DNA from swab samples. We followed the protocol provided in the kit with some modifications; we used Pathogen Lysis Tubes S (#19091, Qiagen). For oral swabs: the samples were collected into 100ul Tris-EDTA buffer (1xTE) and were diluted to 1 ml PBS before the extraction. For humal milk: before the extraction the samples were centrifuged at 4° C *800 g* for 10 min. The phase between the fat coat and pellet was used for extraction. The DNA from all samples was eluted in 30 ul MQ water. The DNA concentrations were measured using Qubit 1X dsDNA HS Assay kit (Thermo Fisher Scientific Inc., USA) on Qubit 3.0 fluorometer (Thermo Fisher Scientific Inc., USA). RNAse was added to each sample (final concentration of 1 µg/µl).

### From meconium

The QIAamp PowerFecal^®^ Pro DNA Kit (#51804; Qiagen GmbH, Germany) was used to isolate bacterial genomic DNA from meconium samples. We followed the protocol provided in the kit with few modifications; 150–250 mg of meconium sample was diluted in 650 µl of solution CD1 before vortexing. Thirty-five µl of Solution C6 was used to elute the DNA. The DNA concentrations were measured using Qubit 1X dsDNA HS Assay kit (Thermo Fisher Scientific Inc., USA) on Qubit 3.0 fluorometer (Thermo Fisher Scientific Inc., USA).

## From infant fecal samples at 1 month of age

As previously [3], eighty to 160 mg of frozen stool was transferred to Precellys soil grinding SK38 lysing tubes (Bertin Technologies, Montigny-le-Bretonneux, France) and one volume of warm (37° C) lysis buffer [4% (w/v) SDS, 50 mM TrisHCl pH 8.0, 500 mM NaCl, 50 mM EDTA] was added. Samples were homogenized for 10 min in room temperature, using a Vortex adapter. Lysozyme (Sigma Aldrich Chemie GmbH, Germany; final concentration 6.25 mg/ml) was added and samples were incubated at 37° C for 30 min, then transferred to 80° C heating block and incubated for 15 min by inverting every 5 min. Samples were then centrifuged at 4° C *20*,*000 g* for 5 min and supernatants were collected; proteinase K (Roche Diagnostics GmbH, Germany) was added (final concentration 0.4 mg/ml) and the samples were incubated, on a heating block, at 70° C for 10 min. After incubation, 10 M NH_4_OAc (final concentration 2 M) was added and samples were incubated on ice for 5 min, then centrifuged at 4° C *20*,*000 g* for 10 min. Supernatants were collected and an equal volume of cold isopropanol was added. The samples were stored on ice for 30 min and thereafter centrifuged at 4° C *20*,*000 g* for 20 min. Pellets were washed 2–3 times with cold 70% ethanol, dried and dissolved overnight in Tris-EDTA buffer (1xTE). The DNA concentrations were measured using Qubit dsDNA Broad Range Assay kit (Thermo Fisher Scientific Inc., USA) on Qubit 3.0 fluorometer (Thermo Fisher Scientific Inc., USA). RNAse was added to each sample (final concentration of 1 µg/µl).

### From oral swabs in newborns and infants at 1 month of age

The GenElute Bacterial Genomic DNA Kit (#NA 2120, NA 2110; Sigma Aldrich, St Louis, US) was used to isolate bacterial DNA from oral swab samples. This kit was selected for infant oral samples due to its reported efficiency in low-biomass conditions, as supported by prior paediatric oral microbiota studies [22]. We followed the manufacturer’s protocol with some modifications; we used lysozyme (#L6876, Sigma). Before the DNA isolation we had prepared the column with 200 µl of Column Preparation Solution. DNA was eluted in 100 µl of the Elution Solution, however we incubated the column at RT for 30 min before the centrifuge. The DNA concentrations were measured using Qubit 1X dsDNA HS Assay kit (Thermo Fisher Scientific Inc., USA) on Qubit 3.0 fluorometer (Thermo Fisher Scientific Inc., USA). RNAse was added to each sample (final concentration of 1 µg/µl).

### 16S rRNA gene library preparation, sequencing, bioinformatics and statistics

The sequencing library was prepared according to Earth Microbiome Project’s Protocol [23] with the following modifications: the fused primers were modified to contain barcode sequence on both forward (341 F) and reverse (805R) primer and selected to target the V3-V4 region instead of the V4 region. The PCR reactions for library preparation were carried out as follows: 20 ng of template DNA or 10 µl of DNA-elute (if concentration was too low to measure) was mixed with 5PRIME HotMasterMix (Quantabio, USA) consisting of: 1U Taq polymerase, 45 nM Cl, 2.5 mM Mg2^+^, 0.2 mM of each dNTP, 0.2 µM of each primer (Eurofins Genomics, Germany) and 0.64 ng bovine serum albumin (BSA) in a final volume of 25 µl per reaction. The PCR conditions were 90° C for 15 s and 94° C for 3 min followed by 35 cycles of 94° C for 45 s, 50° C for 1 min and 72° C for 1.5 min, after which a final elongation step at 72° C for 10 min was performed. For each PCR plate, we included a negative control (non-template water) and a positive control (mock microbial community; Genomic DNA from Microbial Mock Community B (Even, Low Concentration), v5.1 L, for 16 S rRNA Gene Sequencing, HM-782D, obtained from the NIH Biodefense and Emerging Infections Research Resources Repository, NIAID, NIH as part of the Human Microbiome Project). These controls were processed in parallel with the study samples and sequenced alongside them to monitor potential contamination and assess sequencing quality. The DNA concentrations were measured using Qubit 1X dsDNA HS Assay kit (Thermo Fisher Scientific Inc., USA) on Qubit 3.0 fluorometer (Thermo Fisher Scientific Inc., USA). Then libraries were pooled in equimolar concentrations and the amplicon pool was purified according to the protocol using AMPure XP beads (Beckman Coulter, USA). Thirteen libraries were prepared. Prior to amplicon sequencing the amplicon pool was diluted in 10 mM Tris-HCl (pH 8.5) to a final concentration of 4 nM. Following the Illumina recommendations, the amplicon pool was denaturated using an equal amount of 0.2 M NaOH (BioUltra) (Sigma Aldrich Chemie GmbH, Germany) and further diluted to 12 pM (in hybridization buffer (HT1 buffer included in the Reagents Kit v3, Illumina, USA). The pool was finally spiked with 5% denaturated PhiX control library (Illumina, USA). The sequencing was performed using the MiSeq sequencing platform with the Reagents Kit v3, 600 cycles (Illumina, USA).

Sequence read data were demultiplexed using deML v1.1.11 [24], followed by quality filtering with q2-demux and denoising using the DADA2 plugin [25] within the QIIME2 environment (v2024.2) [26]. Sequencing included negative (water blanks) and positive (mock community) controls, which were processed alongside study samples. These controls were excluded from downstream statistical analyses but used to monitor contamination and sequencing accuracy. No ASVs were removed solely on the basis of presumed contamination in order to avoid over-filtering true biological signals. Feature and representative sequence tables were merged using qiime feature-table merge-seqs and qiime feature-table merge-table.

The initial dataset comprised 13,441 amplicon sequencing variants (ASVs) and 35,833,734 demultiplexed sequence reads across 1,945 samples, as inferred by DADA2 in QIIME2. After importing into R v4.3.2, ASVs with zero total counts were removed, yielding 12,268 ASVs in the final dataset. Post-filtering sequencing depth ranged from 0 to 192,352 reads per sample (mean: 17,294; median: 12,595; IQR: 4,007–26,570). Sequencing depth varied across sample types: mother rectal swab (mean: 15,456; median: 10,234), partner rectal swab (mean: 20,107; median: 15,706), meconium (mean: 11,080; median: 9,601), infant stool sample (mean: 26,826; median: 24,269), mother oral swab (mean: 19,388; median: 14,968), partner oral swab (mean: 19,169; median: 16,448), newborn oral swab (mean: 12,251; median: 9,118), infant oral swab (mean: 19,908; median: 13,398), mother vaginal swab (mean: 20,043; median: 15,692), and human milk (mean: 1,958; median: 489). These data were used in all downstream analyses.

ASVs were classified using DADA2’s assignTaxonomy function against the GTDB database v214.0 [27]. Taxonomy was reported at the species level only when supported by high-confidence matches and ecological plausibility.

### Diversity and ordination analysis

α-diversity (Shannon Index) was calculated per sample. Between-group differences were tested using Wilcoxon rank-sum tests, with false discovery rate (FDR) correction applied for multiple comparisons.

β-diversity was evaluated using Bray–Curtis dissimilarity and tested using PERMANOVA (adonis2, vegan package). Within-group dispersion was assessed using PERMDISP (betadisper). For ordination, Principal Component Analysis (PCA) was performed on centered log-ratio (CLR) transformed genus-level data, enabling interpretation of major gradients and associated taxa.

To visualise group-level differences in microbial community composition, Principal Coordinates Analysis (PCoA) based on Bray–Curtis dissimilarity was performed. Clustering patterns were evaluated using PERMANOVA, with corresponding p-values reported to assess statistical significance between delivery-mode and timepoint combinations.

### Network analysis

We constructed Bray–Curtis-based co-occurrence networks using make_network() from phyloseq v1.50.0 [28], setting max.dist = 0.3. Networks visualized ecological clustering across niches and delivery groups.

### Differential abundance analysis

Group-enriched taxa were identified using diff_analysis() from the MicrobiotaProcess [28]. This method applies a two-step non-parametric test procedure: a Kruskal–Wallis test to assess overall group differences, followed by Wilcoxon rank-sum tests for pairwise comparisons, and Linear Discriminant Analysis (LDA) to estimate effect sizes and rank taxa. Taxa were considered significantly enriched in a given group if they met al.l of the following thresholds: Kruskal–Wallis *p* < 0.05 (FDR-adjusted), Wilcoxon *p* < 0.01, and LDA score >3.0. To visualise taxonomic relationships among enriched taxa, cladograms were generated using the ggdiffclade(). The false discovery rate (FDR) adjustment for the Kruskal–Wallis test was performed using the Benjamini–Hochberg method.

### Putative microbial source overlap analysis

To explore potential microbial overlap between infant and adult samples, we focused on ASVs identified as significantly different in infant gut and oral samples according to delivery mode. These ASVs were visualised using a tree-based approach implemented via the plot_tree() function in the phyloseq package. In this plot, nodes represent individual ASVs, and tips are annotated by sample type. The layout integrates phylogenetic relationships with sample-wise ASV presence and relative abundance, enabling visual inspection of co-occurring ASVs across niches, but does not resolve strain-level transmission.

### Demographic analyses

Group differences in demographic variables were tested using the Mann-Whitney U test or Test of Equal or Given Proportions.

### Software and packages

All downstream statistical and visualisation analyses were conducted in R v4.4.2 (R Core Team) [29] using the following R packages: phyloseq v1.50.0 [30], MicrobiotaProcess v1.19.0 [28], microViz v0.12.6 [31], vegan v2.6–10 [32], and ggplot2 v3.5.2 [33]. Unless otherwise specified, statistical significance was defined as FDR-adjusted *p* < 0.05.

## Results

### Study cohort

We recruited 264 pregnant women, 261 partners and 266 infants (Table 1). The mean (SD) age at the time of inclusion, i.e. at the time of the routine ultrasound examination of the pregnant women was 32 (±4.3) years and the corresponding age for their partners was 34 (±5.5) years. Most of the parents were born in Sweden (*n* = 228 and *n* = 214, respectively). For vaginal deliveries, gestational age at delivery was 39.8 ± 1.1 weeks (median 40; IQR 39.0–41.0; range 37.0–42.0), and for CS deliveries it was 39.5 ± 1.3 weeks (median 40; IQR 39.0–41.0; range 37.0–42.0). 

As presented in Table 1, the cohort predominantly included healthy pregnant women, with few cases of gestational diabetes (*n* = 6) and urinary tract infections treated with antibiotics (*n* = 2). Two women reported smoking. Among the pregnant women, 88% (*n* = 232) provided vaginal swabs, 93% (*n* = 242) provided rectal swabs, and 90.5% (*n* = 239) provided oral swabs, all self-sampled on the day of admission to the delivery unit. A proportion of 61.4% (*n* = 162) contributed with a human milk sample at the time when the infant reached 1 month of age. Among the partners, 86.5% (*n* = 226) contributed with rectal and 90% (*n* = 235) with oral swabs, both self-sampled on the day of admission to the delivery unit of the pregnant woman. All 266 infants were born full term, and the majority (82%) were delivered vaginally. Twenty-four newborns (9.0%) were admitted to the neonatal unit, and 8 of them (3%) received antibiotic treatment due to infection (See supplemetary table NEO for diagnosis). While the majority of infants (98%) were breastfed at 1 month of age, 198 (74%) were exclusively breastfed. Additionally, 23 had received some additional formula supplementation during the first week after birth. Within 48 h after delivery, meconium (first pass stool) samples were collected from 91% (*n* = 242) and oral swabs from 83% (*n* = 221) of the newborns. At 1 month of age, stool samples and oral swabs were collected at home from 81% of infants (*n* = 215 for stool and *n* = 214 for oral swabs, respectively) (Table 1). Across the ten planned sample types, 19 families contributed complete sample sets.

**Table 1:** Cohort description

	*Mother* (*n* = 264)	*Partner* (*n* = 261)
Sex (woman)	264	6
Age (years)	32 (± 4.3)	34 (± 5.5)
Weight (kg)^a^	68 (± 12)	84 (± 13)
Height (cm)^b^	167 (± 7)	181 (± 6.8)
Body mass index	24 (± 4)	
Gestational diabetes	2 (0.8%)	
Antibiotic treatment during pregnancy	6 (2%)	
Number of children in the family	1.5 (± 0.7)	
Sweden as country of birth	228 (83%)	214 (82%)
Education (University or College)	209 (79%)	154 (59%)
Smoking habits^*^:		
No smoking before or during pregnancy	254 (96%)	248 (95%)
Current smoker	2 (0.75%)	1 (0.4%)
	***Infant(n = 266)***	
Sex (girl)		125 (47%)
Gestational age (weeks)		40 (± 1.5)
Delivery mode:		
Vaginal delivery (VD)		219 (82%)
Caesarean section (CS)		47 (18%)
Elective CS/Emergency CS		11/36
Birth weight (g)		3583 (± 478)
Birth length (cm)		50 (± 2)
Birth head circumference (cm)		35 (± 1.3)
Apgar score 1´		8.5 (± 0.9)
Apgar score 5´		9.2 (± 0.75)
Apgar score 10´		9.6 (± 0.6)
Admitted to neonatal ward		24 (9%)
Treated with antibiotics		8 (3%)
Breastfeeding at 4 weeks post partum:		
Vaginal (VD) (*n* = 219)		214 (98%)
Caesarean section (CS) (*n* = 47)		46 (99%)

^a, b^Variables recorded at the time of maternal inclusion in the antenatal care program in primary care. Data is presented as mean ± SD and n (%)

^*^Smoking habits were assessed as: no smoking before or during pregnancy, smoking during the last months before pregnancy, or active smoking during pregnancy

### Microbial community structure across families and niches

**Fig. 2:**
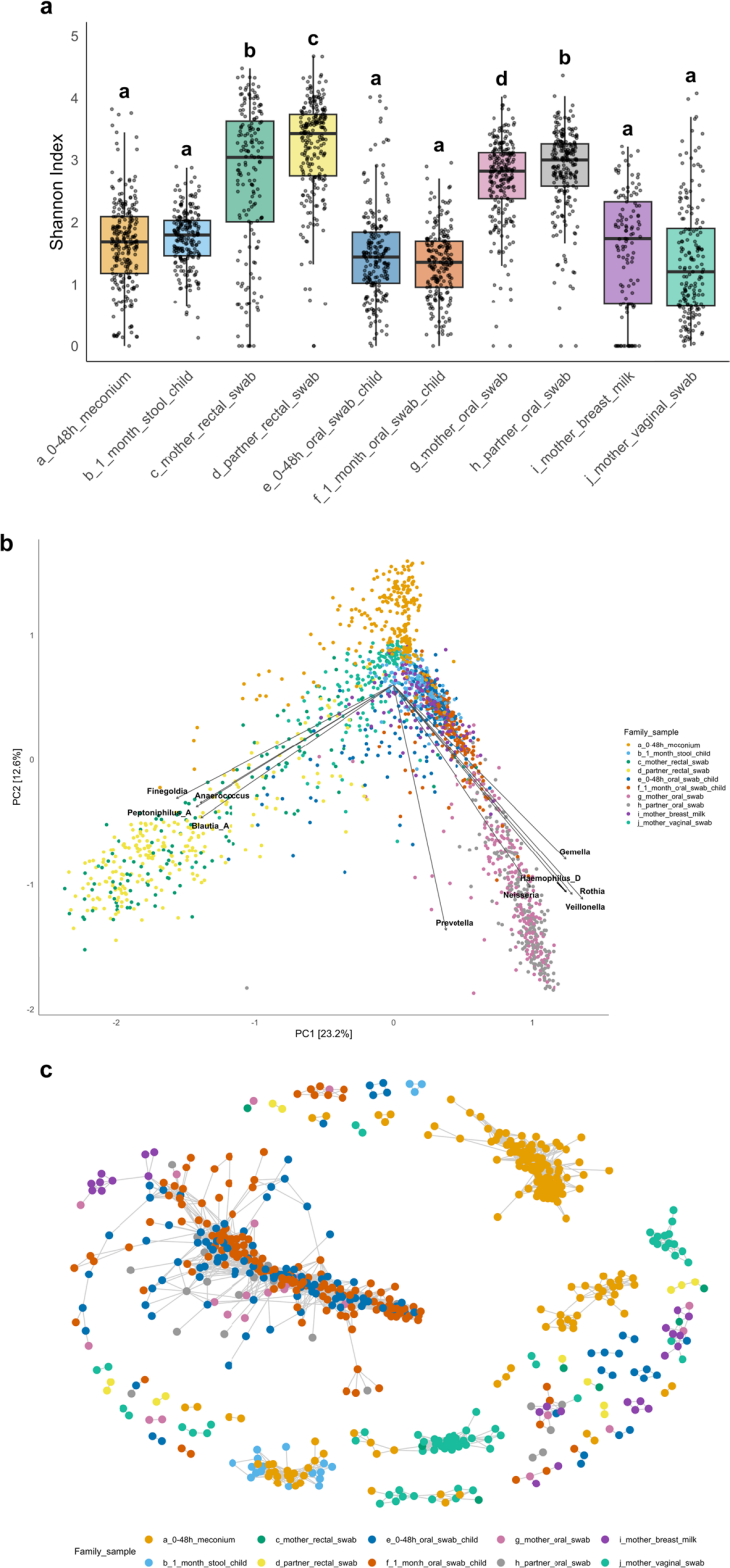
Microbial community structure, and niche-specific relationships across the sample types. **a** α-diversity (Shannon Index) across all sample types. Adult gut and oral samples show significantly higher diversity compared to corresponding newborn and infant samples. Pairwise group comparisons were assessed using Wilcoxon tests with false discovery rate (FDR) correction. Differences between the groups are presented using compact letter display (CLD): groups sharing a letter do not differ; groups without a letter in common differ significantly (FDR-adjusted p < 0.05). Full statistics are provided in Supplementary Table AD. **b** Principal Component Analysis (PCA) on centered log-ratio (CLR)-transformed genus-level data reveals clear niche-related clustering of microbial communities, with strong separation between gut, oral, and vaginal samples. However, samples within each niche (e.g., mother vs. partner, or child vs. adult) clustered closely, indicating similar community structures. Biplot arrows display the top 10 taxa by loading magnitude; notable contributors include gut-associated Finegoldia, Anaerococcus, Blautia_A, and oral swab-associated Neisseria, Gemella, Haemophilus_D. **c** Co-occurrence network based on Bray–Curtis dissimilarities (max.dist = 0.3) shows strong ecological clustering by niche. Distinct and densely connected subnetworks are visible for oral samples (across age groups) and meconium samples, suggesting early-life oral and gut environments already exhibit niche-specific microbial networks.

As shown in Fig. 2a, α-diversity was significantly higher in adults compared to the newborn and infant samples, both in the gut and oral niches (Wilcoxon test, *p* < 0.001; Supplementary Table AD). The PCA plot (Fig. 2b), based on CLR-transformed genus-level data, revealed clear separation between gut, oral, and vaginal microbiota, with some overlap between human milk and oral samples. Niche-specific taxa contributing to this separation are shown as loading vectors (e.g., *Finegoldia*, *Anaerococcus*, and *Blautia_A* for rectal swabs; *Neisseria*, *Gemella*, and *Haemophilus D* for oral swabs).

β-diversity demonstrated significant differences across all pairwise comparisons (PERMANOVA, *p* = 0.0045, Supplementary Table PX), except between mother and partner oral swabs (PERMANOVA, *p* = 0.295). Rectal swabs from mothers and partners differed significantly (PERMANOVA, *p* = 0.001, R² = 1.68%), the β-dispersion analysis showed significantly different within-group variability (PERMDISP, *p* = 0.001; Supplementary Table PY). Microbial similarities between mothers and partners are further illustrated in Supplementary Figs. YSa (gut profiles) and YSb (oral profiles). The co-occurrence network (Fig. 2c), based on Bray-Curtis dissimilarities, underscores ecological specificity, with distinct and well-connected clusters observed for oral swabs, meconium samples, and vaginal swabs.

**Fig. 3:**
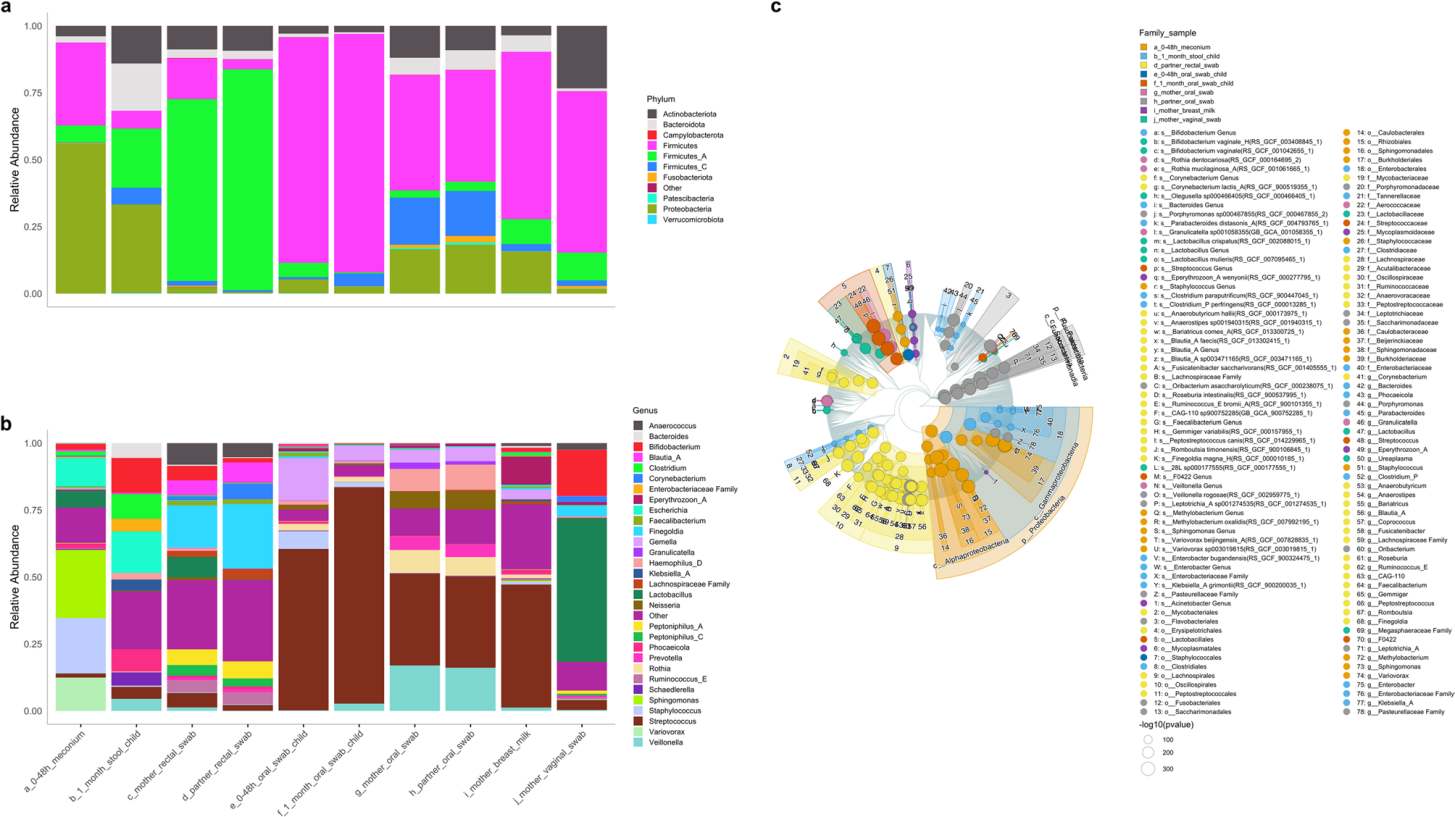
Bacterial composition and niche-specific biomarkers in adult and early-life microbiota. **a**,** b** Relative abundance (RA) of bacterial taxa at the phylum (a) and genus (b) levels across rectal and oral samples from pregnant women and their partners, and corresponding infant samples (meconium and oral swabs within 48 h; fecal and oral swabs at 1 month). RA is shown as the average proportion of ASVs per group. Adult gut and oral samples displayed niche-specific and highly similar microbiota across mothers and partners. Infant gut microbiota initially resembled adult gut profiles at the phylum level but diverged at the genus level, with meconium enriched in *Escherichia* and *Staphylococcus*, and *Bifidobacterium* emerging in fecal samplesat 1 month. Infant oral samples were dominated by *Streptococcus*, reflecting partial continuity with adult oral communities. **c** Cladogram of taxa differentially abundant across all sample groups, generated using diff_analysis() Kruskal–Wallis (FDR-adjusted) *p* < 0.05, Wilcoxon *p* < 0.01, LDA score > 3.0). Each node represents a taxon (from phylum to genus), and node colour indicates the group of enrichment. Circle size reflects statistical significance (-log10 *p* value).

The bacterial composition, presented as proportional relative abundance (Fig. 3a and b), revealed microbial similarity between pregnant women and their partners for both rectal and oral swabs. In contrast, the microbiota of infants differed from adults, both in the relative abundance of shared dominant taxa and in the presence of niche- and time-specific taxa. Meconium samples were enriched in *Escherichia* and *Staphylococcus*, while fecal samples at 1 month of age showed an increased abundance of *Bifidobacterium*. In the oral microbiota, *Streptococcus* was more abundant at 48 h post-partum and at 1 month of age compared to adults.

In the cladogram (Fig. 3c), niche-specific bacterial biomarkers are visualized. Meconium samples were enriched in several genera from Alpha- and Gammaproteobacteria, while fecalsamples at 1 month exhibited an increased relative abundance of *Enterobacteriaceae*, *Bacteroides*, *Parabacteroides*, and *Clostridium_P*. These taxa reflect the transition from early facultative anaerobes toward a more complex gut microbiota in infants over the first month of life. Oral swab samples were enriched in genera such as *Porphyromonas*, *Oribacterium*, and *Leptotrichia_A* for partners; *Granulicatella* for mothers, Staphylococcales for newborns, and *Streptococcus and Lactobacillales* for infants. Microbial taxa specific to vaginal samples included *Bifidobacterium vaginale* (also historically referred to as *Gardnerella vaginalis* (NCBI taxonomy)) and *Lactobacillus crispatus*.

### Mode of delivery and gut microbiota diversity from birth to one month

**Fig. 4:**
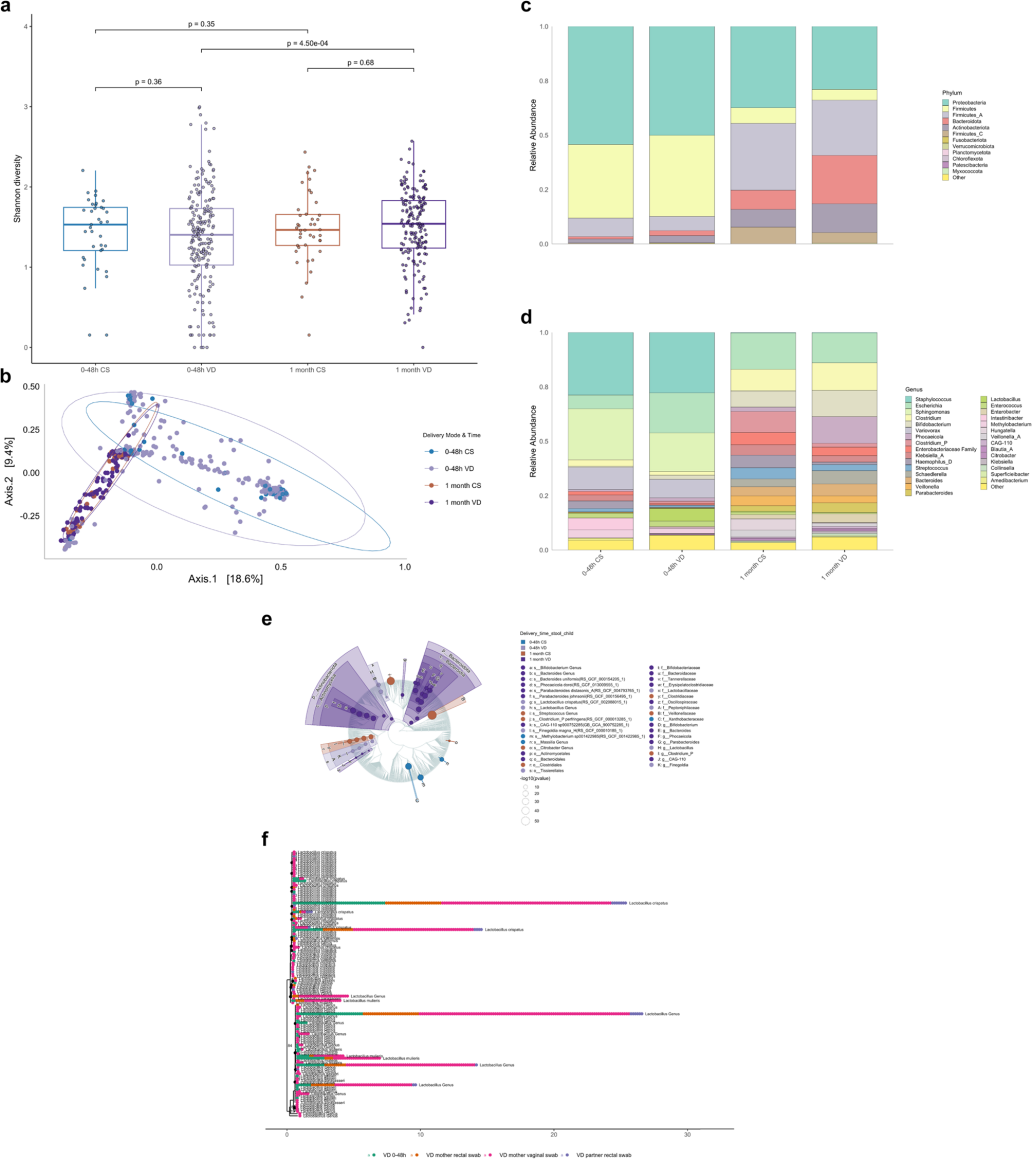
Delivery mode shapes early-life gut microbiota community structure and biomarker profiles. **a** α-diversity (Shannon index) at birth (meconium) and one month of age in vaginally delivered (VD) and Caesarean section (CS) delivered infants. No significant differences were observed between VD and CS delivered infants at either time point. However, α-diversity increased significantly in VD infants between birth and one month (Wilcoxon test, *p* < 0.001), indicating microbiota maturation. **b** Principal Coordinates Analysis (PCoA) plots based on Bray–Curtis dissimilarity, comparing gut microbiota composition in meconium and one-month fecalsamples between vaginally delivered (VD) and Caesarean section (CS) delivered infants. Group-level separation indicates delivery-mode-specific microbial profiles for meconium (PERMANOVA: *p* = 0.038) and borderline significant differences for one-month fecal samples (PERMANOVA: *p* = 0.057). No significant differences in within-group dispersion were observed (PERMDISP: *p* = 0.46 for meconium; *p* = 0.58 for one month fecal sample). **c**,** d** Relative abundance plots at the phylum (c) and genus (d) level show temporal shifts in community composition from birth to one month. While both delivery groups shared dominant phyla (e.g., *Proteobacteria*, *Firmicutes*, *Bacteroidota*), early genera such as *Sphingomonas* and *Staphylococcus* decreased over time, indicating reduced influence of hospital-associated taxa. **e** Differential abundance analysis identified key taxa associated with each delivery mode. Vaginally delivered (VD) infants were enriched in *L. crispatus* and *F. magna* at birth, and in *Bifidobacterium*, *Bacteroides*, and *Parabacteroides* at one month. Caesarean section (CS) delivered infants were enriched in *Methylobacterium* species at birth, and *Streptococcus* species and *C. perfringens* at one month. Taxa were considered significantly enriched based on a two-step non-parametric test (Kruskal–Wallis followed by Wilcoxon rank-sum tests), with FDR-adjusted *p* < 0.05 and LDA score > 3.0.). **f** Shared amplicon sequence variants (ASVs) of *L. crispatus* across maternal and infant niches, visualised using a phylogenetic tree layout. In meconium, the prevalence of *L. crispatus* was 46/219 (21.0%) in vaginally delivered (VD) infants and 2/47 (4.3%) in Caesarean section (CS) infants; only VD infants are shown here to align with the maternal vaginal samples. Nodes represent ASVs, and tip annotations indicate sample types, with relative abundance mapped to node size. This visualisation illustrates potential ecological overlap between maternal vaginal microbiota and neonatal gut colonisation in VD infants. However, interpretations are limited by the resolution of 16S rRNA gene sequencing and should not be considered conclusive evidence of vertical transmission.

As shown in Fig. 4a, α-diversity did not differ significantly between VD and CS delivered infants at either time point. VD infants exhibited a significant increase in α-diversity from birth to one month (Wilcoxon test, *p* < 0.001), whereas this change was not observed in CS delivered infants. β-diversity, assessed using Bray–Curtis dissimilarity and visualised by PCoA (Fig. 4b), revealed significant differences in microbial composition between VD and CS delivered infants for meconium (PERMANOVA: *p* = 0.038) and a borderline significant difference for one-month fecal samples (PERMANOVA: *p* = 0.057). These differences were not accompanied by significant differences in dispersion (PERMDISP: *p* = 0.46 for meconium; *p* = 0.58 for one-month samples, respectively).

### Mode of delivery and gut microbiota taxonomic composition from birth to one month

Relative abundance profiles at the phylum and genus levels (Fig. 4c–d) showed similar proportions of major phyla at around 48 h post-birth in both groups. Hospital-associated genera such as *Sphingomonas* and *Staphylococcus* [34, 35] were more prominent in meconium and decreased by one month in both groups regardless of delivery mode. Differential abundance analysis (Kruskal–Wallis (FDR-adjusted) *p* < 0.05; Wilcoxon *p* < 0.01; LDA score >3.0) revealed delivery-specific microbial signatures (Fig. 4e). In meconium, *L. crispatus* and *Finegoldia magna* were enriched in VD infants, whereas *Methylobacterium sp.* was more abundant in CS delivered infants. To contextualize these findings, we visualised shared *L. crispatus* ASVs in a phylogenetic tree layout across meconium and parental swabs (Fig. 4f). In meconium, *L. crispatus* was detected in 46/219 (21%) VD infants and 2/47 (4.3%) CS-infants. Given the strain-level limits of 16 S rRNA gene sequencing, these observations indicate ecological overlap rather than demonstrating vertical transmission. At one month, VD infants stool was enriched in *Bifidobacterium*, *Bacteroides*, and *Parabacteroides*, whereas *Streptococcus sp.* and *Clostridium perfringens* were more common in CS delivered infants (Fig. 4e).

### Mode of delivery and oral microbiota diversity in early life

**Fig. 5:**
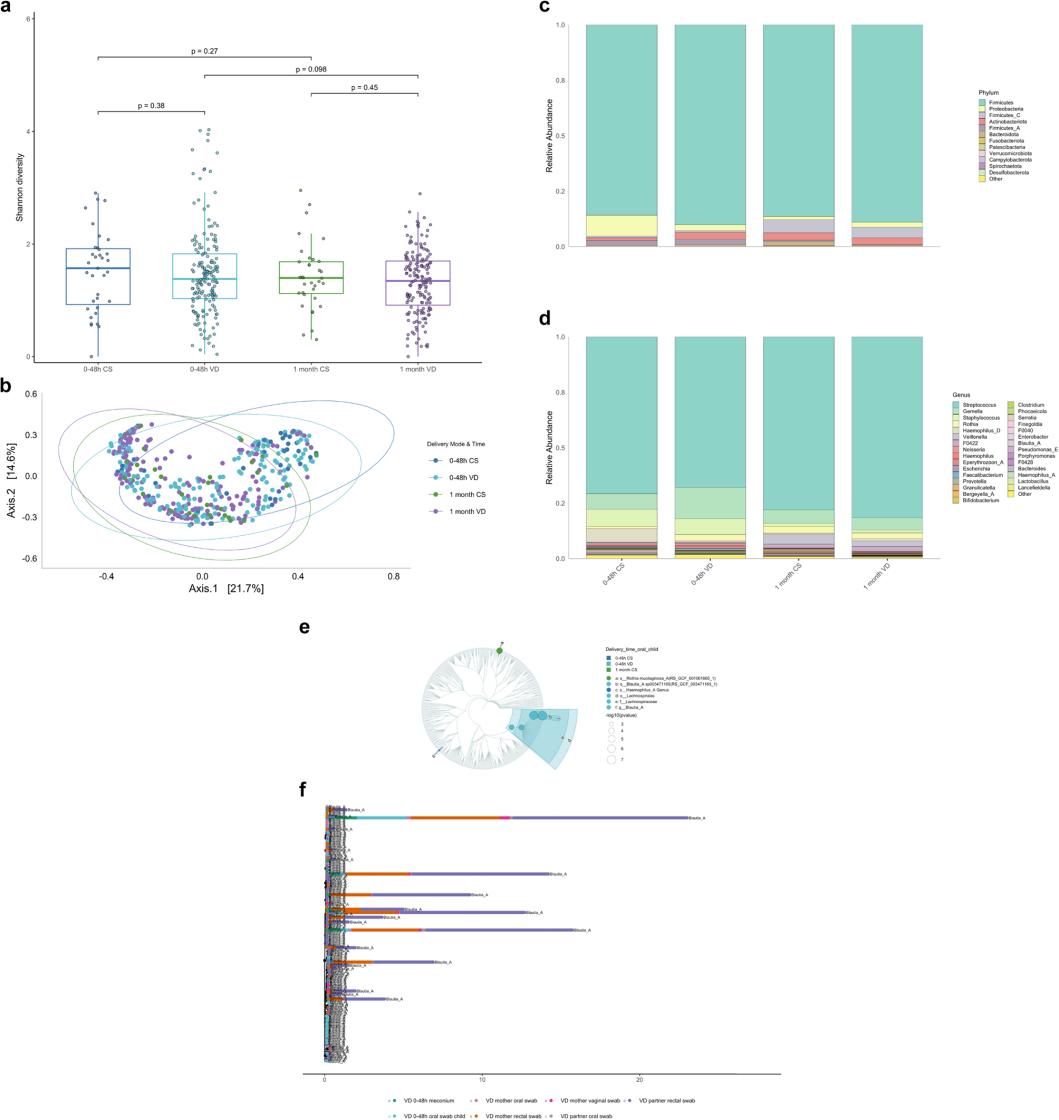
Impact of delivery mode on early-life oral microbiota community structure and taxonomic composition. **a** α-diversity (Shannon Index) of oral microbiota does not differ significantly between vaginally delivered (VD) and Caesarean section (CS) delivered infants at 0–48 h or at one month of age (Wilcoxon test). **b** β-diversity (Bray–Curtis dissimilarity), visualised using a PCoA plot, shows significant differences in oral microbiota composition between VD andCS delivered infants within 48 h of birth (PERMANOVA, *p* = 0.003). At one month of age, this difference was no longer statistically significant (*p* = 0.16). **c**,** d** Relative abundance of bacterial taxa at phylum (c) and genus (d) levels in oral swabs from VD and CS delivered infants. Stacked barplots display average taxonomic profiles per group. **e** Cladogram highlighting *Blautia_A* as significantly enriched in VD infants at 0–48 h. This genus, typically associated with the gut, may indicate early cross-niche exposure. Statistical testing was performed using Kruskal–Wallis and Wilcoxon tests with FDR adjustment (*p* < 0.05) and an LDA score threshold > 3.0. **f** Shared amplicon sequence variants (ASVs) of *Blautia_A* across maternal and infant niches, visualised using a phylogenetic tree layout (plot_tree function, *phyloseq*). In newborn oral swabs, prevalence of *Blautia_A* was 57/219 (26%) in VD infants and 6/47 (13%) in CS delivered infants; only VD infant oral swabs are shown here to align with the maternal rectal samples. Nodes represent ASVs, and tip annotations indicate sample types, with relative abundance mapped to node size. This plot illustrates ecological overlap potentially resulting from vaginal delivery and transient exposure to maternal gut-associated taxa.

When comparing α-diversity between delivery modes at each timepoint, no significant differences were observed (Wilcoxon test, *p* > 0.05, Fig. 5a). β-diversity (Fig. 5b) revealed significant compositional differences at about 48 h (PERMANOVA, *p* = 0.003), but not at one month (*p* = 0.16). Dispersion did not differ significantly at either timepoint (PERMDISP, *p* = 0.28 at 48 h, *p* = 0.07 at one month, respectively).

#### Mode of delivery and oral microbiota taxonomic composition in early life

Relative abundance plots at phylum and genus level (Fig. 5c–d) indicated subtle taxonomic shifts over time and by delivery group. For infants with paired samples, within-group changes from 0 to 48 h to 1 month were tested separately in CS and VD using paired Wilcoxon tests on CLR-transformed abundances at both the phylum and genus levels; no taxa remained significant after FDR correction. Differential abundance analysis (Fig. 5e) identified *Blautia_A* as significantly enriched in oral swabs from VD infants within the first 48 h (Kruskal–Wallis *p* < 0.05 (FDR-adjusted), Wilcoxon *p* < 0.01, and LDA score > 3.0). To contextualise this, we visualised shared *Blautia_A* ASVs in a phylogenetic tree layout across VD newborn oral and parental rectal swabs (Fig. 5f). In newborn oral swabs, *Blautia_A* was detected in 57/219 (26%) VD infants and 6/47 (13%) CS infants. Given the strain-level limits of 16S rRNA gene sequencing, these observations indicate ecological overlap rather than demonstrating vertical transmission.

## Discussion

In this explorative study within a population-based birth cohort, we analysed the gut and oral microbiota in mother-partner-infant triads to map community structures and explore ecological overlap across niches and timepoints. As expected from previous studies [11, 36, 37], the GI microbiota of infants differed from adults not only in the relative abundance of dominant taxa but also in the presence of niche- and time-specific taxa, highlighting the dynamic nature of early-life microbial colonisation. While pregnant women and their partners shared overall microbiota structure in both rectal and oral samples, subtle but consistent differences were evident, aligning with findings from other population-based datasets [11, 36, 37]. The clear niche separation is consistent with strong ecological structuring by body site from birth onwards. In the oral cavity, maternal and partner profiles were more similar to each other than to infant profiles [37], underscoring that early oral assembly follows a distinct developmental trajectory. Gut profiles also reflected expected ecological succession [11]. Importantly, this study was designed as a descriptive investigation to map microbial community structures and explore potential ecological overlap across niches and timepoints. Given the limitations of 16S rRNA gene sequencing—particularly its inability to resolve strain-level variation—our findings should be interpreted as indicative of shared microbial patterns rather than conclusive evidence of vertical transmission or causality [38–40]. This approach, reflecting real-life variation in delivery mode, feeding, and sample timing, aims to inform and guide future studies employing higher-resolution techniques such as shotgun metagenomics.

Early-life meconium samples were enriched in *Escherichia* and *Staphylococcus*, with *L. crispatus* emerging as a delivery-associated signature taxon in VD newborns. *L. crispatus* is a dominant vaginal commensal in healthy pregnancy [41, 42] and has been consistently linked to maternal–infant microbial continuity at birth [43–45]. We focused on this niche-restricted, well-characterised species because it provides a conservative marker of delivery-related exposure, avoiding the limitations of a global shared-ASV analysis that would be underpowered in our cohort and constrained by the strain-level resolution of 16S rRNA sequencing. Its detection in VD meconium, alongside presence in corresponding maternal vaginal swabs, is consistent with a maternal origin [41] and ecological transfer during birth. By one month of age, *L. crispatus* declined in abundance, and other taxa became differentially abundant according to delivery mode. Stool samples from VD infants showed higher levels of *Bifidobacterium*, *Bacteroides*, and *Parabacteroides*, whereas *Streptococcus sp.* and *Clostridium perfringens* were more common in CS delivered infants. These findings are consistent with previous studies demonstrating reduced *Bacteroides* in CS delivered infants [46, 47]. Several of the enriched taxa, particularly within *Bifidobacterium* and *Bacteroides*, were also present in parental rectal and vaginal swabs or human milk samples, suggesting partial ecological overlap across family-associated microbiota. While this overlap aligns with prior studies reporting microbial similarity within families [39, 48], the taxonomic resolution of 16S rRNA gene sequencing limits conclusions at the strain level. Consistent with prior work [39, 40, 48], our results reinforce the influence of delivery mode on early microbial community structure.

Most studies of early-life microbial colonization have focused primarily on the gut, whereas the oral microbiota remains comparatively underexplored. The oral cavity, however, is one of the first colonized mucosal surfaces after birth and may reflect both maternal and environmental exposures. In this study, we included early oral swabs alongside meconium and stool samples to provide a broader ecological view of microbial acquisition. *Blautia_A*, a genus typically associated with the gut microbiota [49], was unexpectedly more abundant in the oral microbiota of VD newborns compared to CS newborns during the first 48 h of life. *Blautia* ASVs were also detected in meconium and in parental rectal swabs, suggesting potential cross-niche occurrence introduced during birth. We highlight this genus because, despite its primary gut association, its early presence in the oral niche may reflect delivery-related microbial exposure. As with all 16S rRNA-based findings, these observations should be interpreted cautiously given the lack of strain-level resolution. Considering oral samples alongside gut and parental microbiota underscores the value of a multi-niche perspective in neonatal microbiome research and supports the inclusion of oral sampling in future studies to better capture early colonisation dynamics.

Given the exploratory and multi-niche nature of this study, the meconium and oral samples were collected within a 48-hour window postpartum, which aligns with the common definition of early neonatal sampling in the literature [50]. However, as discussed above, early microbial composition—both in the gut and oral cavity—can shift rapidly during this critical window, and the timing of meconium passage is largely dictated by infant physiology and neonatal care routines. This limits control over exact sampling time and may partially explain variability in meconium-derived microbial profiles. As oral swabs are more practical to standardise, they were also collected within this early postnatal timeframe as part of our real-world, descriptive sampling strategy, which prioritised feasibility and breadth of coverage across family members and niches.

In this study, we uniquely included microbiota samples from both mothers and partners, enabling a rare opportunity to explore the broader familial microbial environment. We hypothesized that partners might contribute uniquely to neonatal microbiota, particularly in CS delivered infants, where maternal transmission is limited due to lack of vaginal and rectal contact, and altered skin-to-skin interactions. In our setting, it is common clinical practice for the CS delivered infant to have early skin-to-skin contact with the partner while the mother remains in surgery. This clinical routine further motivated our investigation into potential partner-derived microbial seeding. However, due to the high similarity between mothers and partners microbiota in both gut and oral niches, we were unable to identify partner-specific microbial signatures. This suggests that while partners likely influence early microbial exposures, distinguishing their contributions remain challenging with the current sequencing resolution. A recent metagenomic study reported specific paternal strain contributions approaching those maternal origin by 1 year of age [18], underscoring the need for higher-resolution approaches to resolve such dynamics.

The design of this study, in which families collected samples although supervised by health care professionals, but without strict clinical control, reflects the real-world microbial exposures that shape early colonization. Although this approach introduces some analytical variability, it enhances ecological validity. Such study designs are critical for understanding microbiota development in population-based cohorts and offer a strong foundation for future investigations with more mechanistic and strain-resolved approaches.

A key strength of our study is its prospective, population-based birth cohort design, with recruitment during pregnancy allowing for the inclusion of both maternal and paternal (co-parental) samples - a combination rarely addressed in infant microbiota research. We examined microbial composition across multiple ecological niches, including low-biomass samples such as meconium, human milk, and oral swabs, thereby capturing microbial contributions not assessed in many previous studies. By analyzing two early time points—within 48 h of birth and at 1 month—we achieved high early life resolution in characterising the initial establishment of gut and oral microbiota.

This study has also some limitations. First, the sampling strategy relied on parent-conducted collection. Dropouts were most pronounced among adult participants, likely due to the practical challenges of self-sampling in a clinical environment at the time of delivery. In contrast, infant sampling was more consistent across timepoints. Additionally, some samples were excluded due to technical issues, including insufficient DNA yield, failed amplicon PCR, or inadequate sequencing output, which slightly reduced the total number of analyzable specimens. Second, different bacterial DNA extraction kits were used for different sample types, chosen to optimise yield and quality given differences in matrix composition and biomass (e.g., high-biomass fecal samplesvs. low-biomass oral swabs). While this approach can improve recovery from each matrix, it may also introduce kit-dependent biases in microbial community profiles, as reported in previous studies [51, 52]. We have partially addressed this by performing an internal comparison using adult oral swabs processed with both oral swab protocols, which showed similar results at the genus level. However, this could not be replicated for all sample types due to limited material, and some technical bias between matrices cannot be excluded. Third, all human milk samples were collected at one month postpartum, which may not capture longitudinal microbial dynamics. Samples were collected either manually or using a breast pump, reflecting real-life variability in expression methods. As our aim was to capture the microbial exposures relevant to breastfeeding, including skin-associated taxa, we did not standardize for potential differences in skin microbiota contribution between sampling modes. This may introduce variability in low-abundance taxa, a known consideration in human milk microbiome studies [53]. Fourth, although we used 16S rRNA gene-specific amplification and extraction kits optimized for microbial enrichment, low-biomass samples such as oral swabs and human milk remain susceptible to environmental or reagent contamination. We did not apply in silico decontamination filters, and while contamination risk is likely low, especially for dominant taxa, results should be interpreted with caution—particularly for low-prevalence or low-abundance ASVs. Fifth, while 16S rRNA gene sequencing provided robust community-level and taxonomic profiling, it does not resolve strain-level diversity or functional capacity. Taxonomic assignments were based on the GTDB database (v214.0) and reported at the species level only when supported by ecological plausibility. The observation of shared ASVs between parents and infants should be interpreted cautiously—as these may reflect microbial source overlap rather than confirmed vertical or parental transmission. Strain-resolved metagenomics would be required to robustly assess parental microbial contributions. Sixth, this study captured only the earliest stages of infant gut and oral microbiota colonization, with no follow-up beyond one month of age. As such, it cannot assess long-term microbial succession, persistence of early colonizers, or associations with health outcomes. Finally, we did not perform multivariable modelling to adjust for potential clinical, environmental, or technical covariates. Relevant factors that may influence early microbiota include intrapartum antibiotic exposure, postnatal antibiotics, infant feeding, gestational age and birth weight, infant sex, parity, duration of membrane rupture, and household exposures (siblings, pets) as well as seasonality. Technical covariates were also not modelled. Our design was primarily descriptive, and subgroup sizes limited robust adjustment; therefore, residual confounding cannot be excluded. The ongoing NorthPop study will enable future research to link early GI colonization with health outcomes in childhood.

## Conclusions

Collectively, our findings suggest that delivery mode and parental microbiota influence the very early composition of gut and oral microbiota. Despite the expectation that partners might contribute uniquely to the neonatal microbiota, in this cohort partner and maternal microbiota profiles were highly similar, making it challenging to identify distinct partner-specific influences using 16S rRNA gene amplicon sequencing. These findings come from a population-based, prospective study using 16S rRNA gene amplicon sequencing, which provides valuable insights into early-life microbiota patterns. While this approach offers broad taxonomic coverage, higher-resolution sequencing and expanded covariate analyses in future studies will help to resolve subtle microbial contributions within families and further clarify their potential implications for infant health.

## Supplementary Information

Supplementary Material 1.

Supplementary Material 2.

Supplementary Material 3.

Supplementary Material 4.

## Data Availability

The dataset supporting the conclusion in this article is available in the NCBI Sequence Read Archive (SRA) under the BioProject number PRJNA1206707. Amplicon sequences are provided in FASTQ format with limited metadata. Aggregated data that do not allow identification of individuals are available from the corresponding author upon reasonable request. Correspondence and requests for materials should be addressed to Dr. Christina E. West.

## Abbreviations

VD: Vaginal delivery
CS: Caesarean section
GI: Gastrointestinal
ASV: Amplicon Sequence Variant
SCFA: Short–Chain Fatty Acids
CLR: Centered Log–Ratio transformation
PCA: Principal Component Analysis
PCoA: Principal Coordinates Analysis
LDA: Linear Discriminant Analysis
PERMANOVA: Permutational Multivariate Analysis of Variance
PERMDISP: Permutational Analysis of Multivariate Dispersions
ANOVA: Analysis of Variance
