# Supplementary material for: Early-life gut and oral microbiota development: a multi-niche study including mother-partner-infant triads
Source: BMC Microbiol. 2025 Nov 15;25:751. doi: 10.1186/s12866-025-04521-3 (PMC12619262; doi:10.1186/s12866-025-04521-3)
Supplement: Supplementary file 4 — Supplementary Material 4. [file 12866_2025_4521_MOESM4_ESM.pdf]

Supplementary Figure YS. Microbial dissimilarities (Bray-Curtis) between mothers and partners in stool and oral samples

a

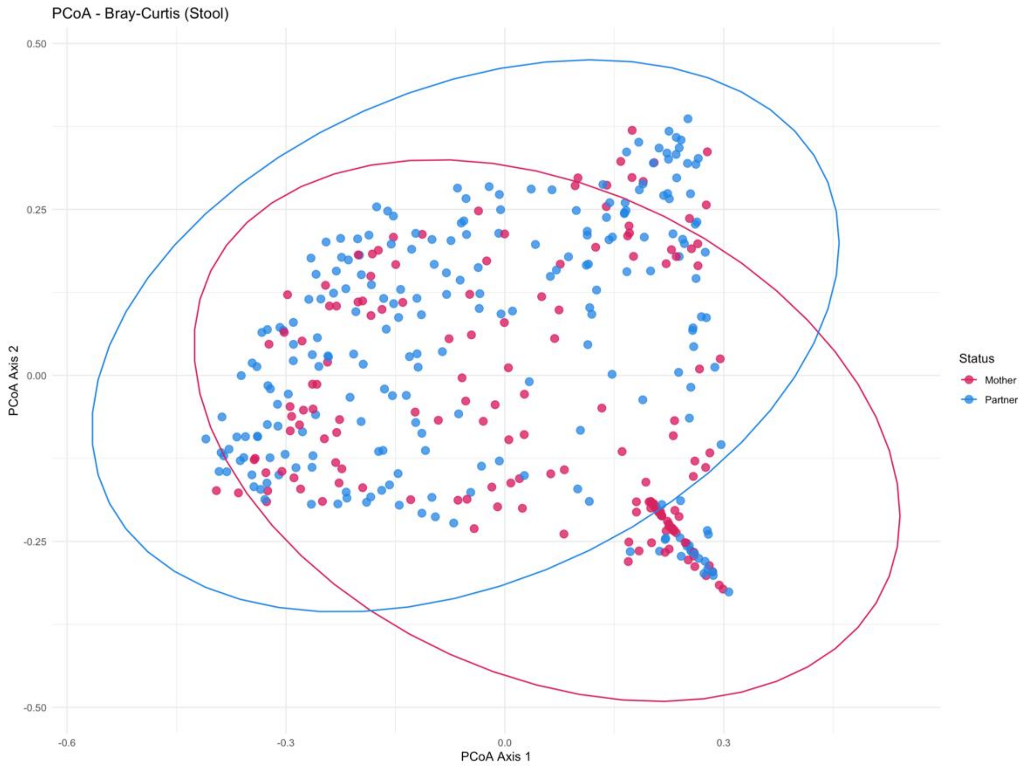

b

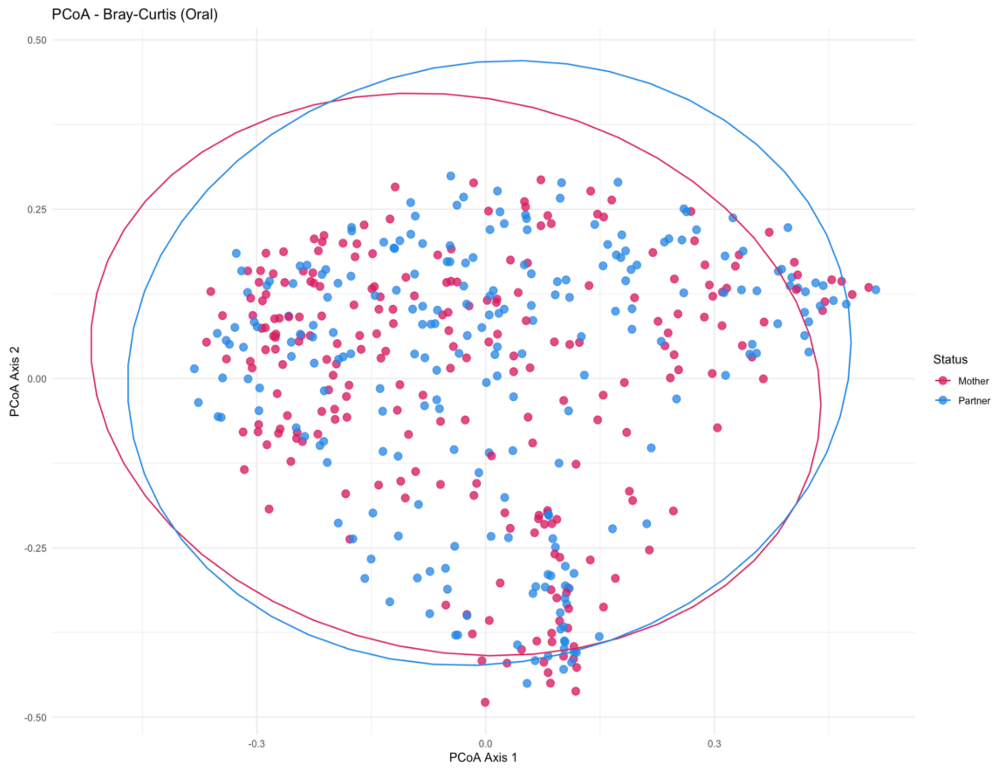

Permutation test for adonis under reduced model
Permutation: free
Number of permutations: 999
Df SumOfSqs R2 F Pr(>F)
Model 1 2.512 0.01681 6.7197 0.001
Residual 393 146.907 0.98319
Total 394 149.419 1.00000

Permutation test for homogeneity of multivariate dispersions
Permutation: free
Number of permutations: 999
Response: Distances
Df Sum Sq Mean Sq F N.Perm Pr(>F)
Groups 1 0.4497 0.44975 51.9 999 0.001
Residuals 393 3.4056 0.00867

Permutation test for adonis under reduced model
Permutation: free
Number of permutations: 999
Df SumOfSqs R2 F Pr(>F)
Model 1 1.083 0.00691 3.2054 0.001
Residual 461 155.799 0.99309
Total 462 156.882 1.00000

Permutation test for homogeneity of multivariate dispersions
Permutation: free
Number of permutations: 999
Response: Distances
Df Sum Sq Mean Sq F N.Perm Pr(>F)
Groups 1 0.0015 0.0015419 0.1711 999 0.692
Residuals 461 4.1553 0.0090137
